# The League of Mercy and the Red Cross

**Published:** 1920-12-25

**Authors:** 


### The League of Mercy and the Red Cross.

Few people realised when the British Red Cross Society began to develop its scheme for collecting sums from small subscribers in aid of the hospitals that it was poaching on the domain of the League of Mercy. But this fact became clear at the annual meeting of the League which took place on Monday, when Princess Alice, Countess of Athlone, occupied the chair as Lady Grand-President in the absence of the Prince of Wales, and delivered an able address. Thanks to the admirable spirit which pervades both these two Societies, all friction has been avoided and the operations of the League and of the Red Cross have been linked together in a joint appeal.

The sum of £25,483 has been collected by the League this year, mostly in small sums and a good deal of it by means of door-to-door collectors. There is still a great deal of improvement in organisation necessary if the collector is not to become a nuisance and thus defeat the aims of charity. The large funds which distribute to every reputable institution within their area are able to avoid the overlapping which tends to unpopularity. We tremble sometimes to hear of competing institutions and societies dividing up their district, quite independently of each other, and engaging kind ladies to visit every house. The plight of the householder thus besieged by a succession of eloquent pleaders is worse than that of the mother with six nurses calling in one morning to improve the health of the family. Some system there should surely be, and the League of Mercy, with the Red Cross as coadjutor, should take things in hand, summon representatives in every town to confer on the right methods to employ, and discourage the incessant calls which stifle benevolence. Most people of limited means who give at all would rather do so at stated intervals than all at once, and every one prefers to have his contribution collected by the same person if possible. It should not be difficult to arrange for a quarterly collection of small sums for the League in a manner which should make it clear that this was the authorised mode of appeal adopted by all the institutions in a particular district or town.

The great service which the League of Mercy has rendered, quite independently of the actual totals collected, has been the encouragement of the giver unable to soar to a guinea, and the development of a fine organisation for securing these small gifts. There can be no doubt among those who have watched the League from its commencement that a vast expansion lies within its reach. Its alliance with the British Red Cross Society should prove stimulating to growth.

